# Synergetic interaction between neighboring platinum and ruthenium monomers boosts CO oxidation†
†Electronic supplementary information (ESI) available. See DOI: 10.1039/c9sc00658c


**DOI:** 10.1039/c9sc00658c

**Published:** 2019-04-25

**Authors:** Peng Zhou, Xingang Hou, Yuguang Chao, Wenxiu Yang, Weiyu Zhang, Zijie Mu, Jianping Lai, Fan Lv, Kuan Yang, Yuxi Liu, Jiong Li, Jingyuan Ma, Jun Luo, Shaojun Guo

**Affiliations:** a Department of Materials Science and Engineering , Peking University , Beijing 100871 , China . Email: guosj@pku.edu.cn; b Center for Electron Microscopy , Tianjin Key Laboratory of Advanced Functional Porous Materials , Institute for New Energy Materials & Low-Carbon Technologies , School of Materials , Tianjin University of Technology , Tianjin 300384 , China; c Key Laboratory of Eco-Chemical Engineering , Taishan Scholar Advantage and Characteristic Discipline Team of Eco Chemical Process and Technology , College of Chemistry and Molecular Engineering , Qingdao University of Science and Technology , Qingdao 266042 , China; d Laboratory of Catalysis Chemistry and Nanoscience , Department of Chemistry and Chemical Engineering , College of Environmental and Energy Engineering , Beijing University of Technology , Beijing 100124 , China; e Shanghai Synchrotron Radiation Facility , Shanghai Institute of Applied Physics , Chinese Academy of Sciences , Shanghai 201204 , China; f The Beijing Innovation Center for Engineering Science and Advanced Technology , Peking University , Beijing 100871 , China; g Key Laboratory of Theory and Technology of Advanced Batteries Materials , College of Engineering , Peking University , Beijing 100871 , China

## Abstract

Sub-nanometer noble metal catalysts, especially single atom (SA), are a new class of catalytic materials for boosting catalysis and possess unique catalytic properties and high atomic utilization efficiency. Exploring the interaction between two neighboring atom monomers has great potential to further improve the performance of SA catalysts and deepen the understanding on the catalytic mechanism of heterogeneous catalysis at the atomic level. Herein, we demonstrate that the synergetic effect between neighboring Pt and Ru monomers supported on N vacancy-rich g-C_3_N_4_ promotes the catalytic CO oxidation. The experimental observation and theoretical simulation reveal that the N vacancy in the g-C_3_N_4_ structure builds an optimized triangular sub-nanometer cavity for stabilizing the neighboring Pt–Ru monomers by forming Pt–C and Ru–N bonds. The mechanistic studies based on the *in situ* IR spectrum and theoretical simulation confirm that the neighboring Pt–Ru monomers possess a higher performance for optimizing O_2_ activation than Ru–Ru/Pt–Pt monomers or isolated Ru/Pt atoms by balancing the energy evolution of reaction steps in the catalytic CO oxidation. The discovery of the synergetic effect between neighboring monomers may create a new path for manipulating the catalytic properties of SA catalysts.

## Abstract 2

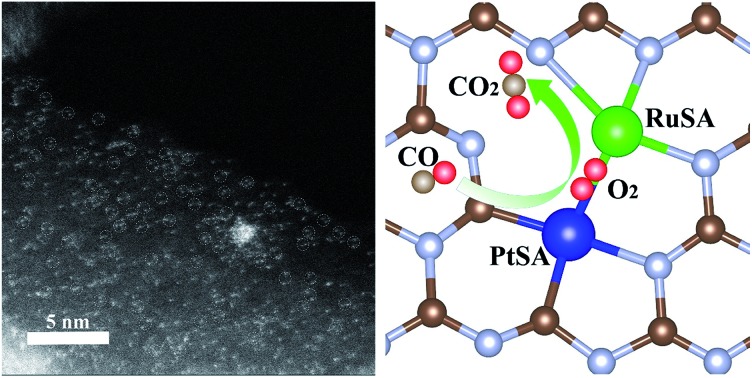
The synergetic effect between neighboring Pt and Ru monomers supported on N vacancy-rich g-C_3_N_4_ promotes the catalytic CO oxidation.

## 

Recently, sub-nanometer single atom (SA) metal catalysts have attracted increasing attention due to their significantly high atom utilization efficiency, low-coordination environment and strong metal–support effect.^1^–^6^ SA catalysts exhibit remarkable activity in a variety of catalytic reactions, including thermocatalytic oxidation, photocatalytic reduction and electrocatalytic hydrogenation.^7^–^12^ More importantly, SA catalysts often follow different catalytic pathways from those of their nanocrystals, leading to product selectivity in catalytic reactions.^9^,^13^–^17^ Furthermore, decreasing the distance between two active SA centers to the sub-nanometer scale has been revealed to have a significant effect on their catalytic behavior.^11^ For instance, isolated Pt monomers favored the conversion of CO_2_ into methanol without the formation of formic acid, whereas CO_2_ was reduced to formic acid on two neighboring Pt monomers.^18^ Moreover, Fe_2_ clusters on g-C_3_N_4_ were confirmed to possess a higher performance for producing active oxygen species than isolated Fe atoms, leading to their unique reactivity for the epoxidation of *trans*-stilbene to *trans*-stilbene oxide.^19^ In those cases, the chemical environments and species of two neighboring monomers are both the same, which limits the diversity of catalytic behavior. Designing two different neighboring monomers probably provides a strategy to reach this target. Furthermore, the lattice limitation from the support often inhibits the formation of two neighboring monomers. Optimizing the surface defect of the support to stabilize the coordination structure of two neighboring monomers is significant for preparing a high-performance catalyst.^4^,^14^,^20^,^21^

Herein, we report neighboring Pt and Ru (Pt–Ru) monomers on N-vacancy-rich g-C_3_N_4_ for the catalytic CO oxidation by an icing-assisted photocatalytic reduction method. X-ray absorption fine structure (XAFS) and high-angle annular dark field-scanning transmission electron microscopy (HAADF-STEM) results confirm that the surface of g-C_3_N_4_ (CN) is covered with the high-density Pt–Ru monomers. The theoretical simulations suggest that the N vacancy in the g-C_3_N_4_ structure builds an optimized triangular sub-nanometer cavity for stabilizing the neighboring Pt–Ru monomers by forming Pt–C and Ru–N bonds. X-ray absorption near-edge structure (XANES) and X-ray photoelectron spectroscopy (XPS) indicate that the Ru monomer and N vacancy synergistically increase the electron density of the neighboring Pt monomer. The catalytic CO oxidation shows that the formation of electron-rich Pt–Ru monomers in the N vacancy region contributes to improved catalytic activity. The mechanism study based on the *in situ* IR spectrum and theoretical simulation suggests that the catalytic CO oxidation on Pt–Ru monomers supported by CN follows the Eley–Rideal (E–R) mechanism, in which the electron-rich Pt–Ru monomers optimize the O_2_ activation by causing bridge-type O_2_ adsorption between Pt and Ru compared to the Ru–Ru/Pt–Pt monomers or the isolated Ru/Pt atoms. The discovery of the synergetic interaction between two neighboring different monomers may create a path for manipulating the catalytic properties of SA catalysts.

In a typical synthesis of C_3_N_4_ nanosheets, pure g-C_3_N_4_ was synthesized by annealing dicyandiamide at 500 °C in air, and denoted as CN.^22^,^23^ Then, N vacancies were introduced into g-C_3_N_4_ by treatment at 620 °C in N_2_, and denoted as CN620. X-ray diffraction (XRD) patterns of CN and CN620 show a diffraction peak at 27.2° (Fig. S1a†), indexed to typical g-C_3_N_4_.^22^,^24^ Besides, the intensity of the diffraction peak at 27.2° in CN620 is weaker than that in CN, implying the weakening of crystallinity and formation of defects. The UV-vis diffuse reflectance spectra (DRS) reveal that a progressive redshift in the absorption edge is achieved from CN to CN620 (Fig. S1b†). The bandgaps of the different CN determined from the transformed Kubelka–Munk function progressively narrow from 2.62 eV for CN to 2.14 eV for CN620 (the inset of Fig. S1b†). This redshift results from the formation of N vacancies.^25^,^26^ In the structure of g-C_3_N_4_, the C atoms are coordinated to three N atoms, while some N atoms are only coordinated to two C atoms (Fig. 1a). According to the structure simulation, the largest vertical dimension in pristine CN is 4.20 Å. However, with the formation of N vacancies, two neighboring two-coordinated C atoms (C_2C_) were left. The largest vertical dimension in N-vacancy-modified CN is remarkably increased to 5.47 Å. This greatly improves the possibility of creating two neighboring monomers, like Pt and Ru atoms with a diameter of approximate 2.2 Å. According to a recent report, the N vacancy in CN is a photogenerated-electron-rich region, which can reduce the precursor ions of noble metals to monomer atoms.^26^

**Fig. 1:**
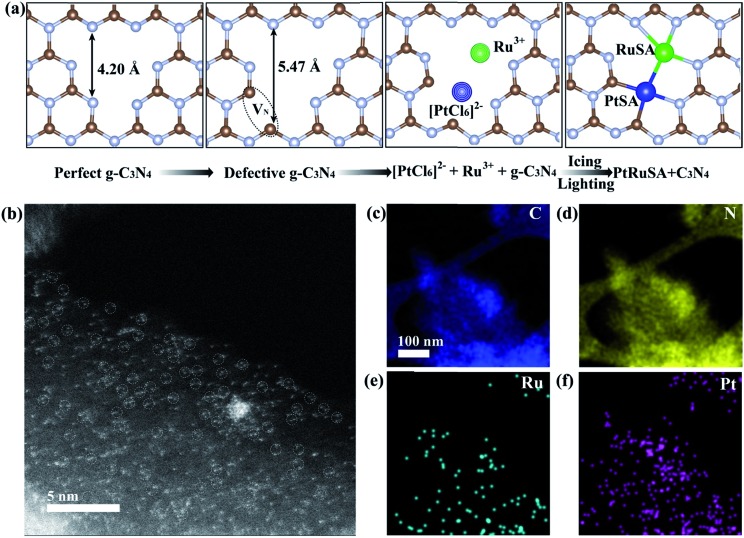
(a) Schematic illustration of the icing-assisted *in situ* photocatalytic reduction method for preparing PtRuSA–CN620. (b) HAADF-STEM images of PtRuSA–CN620 and the corresponding distribution of (c) C, (d) N, (e) Ru and (f) Pt. Scale bar: 5 nm.

Based on the above speculation, Pt–Ru monomer-loaded CN620 (PtRuSA–CN620) was prepared by the method illustrated in Fig. 1a. The CN620 photocatalyst and Pt/Ru precursor (H_2_PtCl_6_ and RuCl_3_) were mixed in deionized water, and then rapidly frozen in liquid nitrogen to form an ice bulk.^27^,^28^ On the CN620 surface, the C_2C_ and N_2C_ sites were positively and negatively charged due to their different electronegativity, respectively. Hence, the negatively charged [PtCl_6_]^2–^ ion and positively charged Ru^3+^ tended to be selectively adsorbed at the C_2C_ and N_2C_ sites, respectively. A 300 W Xe lamp was used as the light source to excite g-C_3_N_4_ in the ice bulk to produce photogenerated electrons. The photogenerated electrons reduced the adsorbed [PtCl_6_]^2–^ and Ru^3+^ ions to Pt and Ru monomers, respectively. RuSA–CN620, PtSA–CN620 and PtRuSA–CN were obtained by a similar method by changing the species of the support and metal ions. For comparison, PtRu nanoparticle-loaded CN620 (PtRuNP–CN620) was prepared by directly irradiated the mixed solution of CN620 and the Pt/Ru precursor without the icing treatment. According to the ICP-OES measurements, the Ru contents in RuSA–CN620, PtRuSA–CN620, PtRuSA–CN and PtRuNP–CN620 are 0.40, 0.45, 0.32 and 0.33 wt%, respectively (Table S1†). The Pt contents in PtSA–CN620, PtRuSA–CN620, PtRuSA–CN and PtRuNP–CN620 are 1.34, 0.51, 0.59 and 0.54 wt%, respectively. These results suggest that the Ru and Pt contents in PtRuSA–CN620, PtRuSA–CN and PtRuNP–CN620 are similar.

Aberration-corrected HAADF-STEM was used to characterize the distribution of PtSA and RuSA on the as-prepared samples. In PtRuSA–CN620, the supra-high-density PtSA and RuSA are observed on the whole CN620 surface (Fig. 1b). Especially, a spacing of approximate 2.8 Å are observed between two neighboring atom monomers, which is consistent with the theoretical structure (Fig. 1a). The corresponding elemental mapping confirms that Pt and Ru share a similar distribution, implying the existence of Pt–Ru monomers (Fig. 1c–f). It should be noted that it is still difficult to distinguish between PtSA and RuSA due to the vibration induced by the irradiation of the electron beam. Similarly, the HAADF-STEM images of RuSA–CN620 and PtSA–CN620 also show high-density RuSA and PtSA, respectively (Fig. S2†). Meanwhile, some Ru–Ru or Pt–Pt monomers are observed. However, the difference is that some remarkable RuNPs with a diameter of larger than 2 nm exist in RuSA–CN620 (Fig. S2a and b†). In contrast, no observable PtNPs appear in PtSA–CN620 (Fig. S2c and d†). Interestingly, the aggregation of RuSAs in PtRuSA–CN620 is effectively inhibited in the presence of PtSAs (Fig. 1b). This implies the interaction between Pt and Ru. This is also observed in PtRuSA–CN though the aggregation degree of Ru is higher than that in PtRuSA–CN620 (Fig. S3†). As a comparison, numerous PtRuNPs are observed in PtRuNP–CN620 (Fig. S4†).

X-ray absorption fine structure (XAFS) measurements were carried out to investigate the coordination structures of Ru and Pt species at the atomic level. Fig. 2a and b show the Ru K-edge and Pt L_3_-edge X-ray absorption near-edge structure (XANES) spectra of the as-prepared samples, respectively. The Ru K-edge absorption thresholds in RuSA–CN, PtRuSA–CN and PtRuSA–CN620 are nearly similar, which are lower than that of Ru foil. Hence, the chemical valences of Ru in the prepared samples are more positive than that of Ru^0^ in Ru foil, but less positive than that of Ru^3+^ in RuCl_3_ according to the previous literature.^10^ The Pt L_3_-edge of PtRuSA–CN and PtRuSA–CN620 is higher than that of PtSA–CN620, but lower than that of Pt foil. This suggests that the existence of RuSA increases the electron density of PtSA in PtRuSA–CN and PtRuSA–CN620. Moreover, the Pt L_3_-edge of PtRuSA–CN620 is higher than that of PtRuSA–CN. Thus the chemical valences of Pt in PtSA–CN620 and PtRuSA–CN is less negative than that in PtRuSA–CN620, but more negative than that of Pt^4+^ in H_2_PtCl_6_ according to the previous literature.^29^ The Fourier-transformed (FT) *k*^3^-weighted extended X-ray absorption fine structure (EXAFS) spectra of Ru further reveal that one main peak at 1.57 Å is observed in RuSA–CN, PtRuSA–CN and PtRuSA–CN620 (Fig. 2c), corresponding to the first coordination shell of Ru.^10^ Similarly, one main peak at 1.60 Å in the FT *k*^3^-weighted EXAFS spectra of Pt corresponds to the first coordination shell of Pt in PtSA–CN, PtRuSA–CN and PtRuSA–CN620 (Fig. 2d).^26^,^29^ Relatively weak peaks ranging from 2 Å to 3 Å are also observed in PtRuSA–CN and PtRuSA–CN620, which are probably attributed to the Ru–Ru or Ru–Pt coordination shell of Ru (Fig. S5†). To obtain the quantitative chemical configurations of RuSA and PtSA, EXAFS fitting was performed to extract the structural parameters (Fig. S6 and S7†). The centered Ru atoms possess three coordinating interactions: Ru–N, Ru–C and Ru–Ru/Pt (Table S2†). It should be noted that it is commonly difficult to distinguish between Ru/Pt–N and Ru/Pt–O coordination due to their similar bond length. Thus the fitted Ru–N coordination probably contains Ru–O coordination. The coordination number of Ru–N in RuSA–CN620, PtRuSA–CN620 and PtRuSA–CN is 7.81, 2.20 and 6.50, respectively. However, the coordination number of Ru–C in RuSA–CN620, PtRuSA–CN620 and PtRuSA–CN is 0.43, 3.48 and 2.58, respectively. This suggests that the introduction of Pt greatly promotes the formation of Ru–C coordination bonds between CN620 and Ru. This is considered to be due to the reason that the Pt-promoted atomic dispersion of Ru leads to the production of more chemical bonds between CN620 and Ru, especially Ru–C. Besides, numerous N vacancies in CN620 also contribute to high coordination numbers of Ru–C in PtRuSA–CN620 due to the more unsaturated C_2C_ atoms. The fitting results of Ru show that the coordination numbers of Ru–Ru (0.36) in PtRuSA–CN620 is smaller than that (1.38) in RuSA–CN620, and meantime, Ru–Pt with a coordination number of 0.72 is obtained, indicating the direct interaction between Pt and Ru. This strongly confirms the existence of numerous neighboring Pt–Ru monomers, which well explains the Pt-promoted atomic dispersion of Ru in the above STEM characterization. However, it should be noted that the higher content of Ru–Pt coordination in PtRuSA–CN is probably originated from the formation of more RuPt alloy clusters or particles according to the above STEM observations (Fig. S3†). This also implies the important role of N vacancies in the formation of neighboring Pt–Ru monomers. Similarly, the existence of Ru also influences the coordination structure of Pt (Table S3†). The coordination number of Pt–C in PtSA–CN620 is decreased from 2.02 to 1.29 in PtRuSA–CN620. Meanwhile the coordination number of Pt–N is also decreased from 5.62 to 3.43, which is attributed to the formation of Pt–Ru bonds with a coordination number of 0.98. Besides, the Pt–C coordination in PtRuSA–CN is negligible due to the fewer N vacancies in CN. Similarly, the high coordination number (1.34) of Pt–Ru can also be attributed to the formation of some RuPt alloy clusters or particles, which is approximate to the fitting results of Ru. The wavelet transform (WT) is used to analyze Ru K-edge (Fig. 2e) and Pt L_3_-edge (Fig. 2f) EXAFS oscillations. The WT maximum of Ru at 4.6 and Pt at 5.6 Å^–1^ could be assigned to the Ru–C/N/O bonding and Pt–C/N/O, respectively. The above XAFS confirms the existence of neighboring Pt–Ru monomers in PtRuSA–CN620 and the important role of N vacancies in the formation of neighboring Pt–Ru monomers.

**Fig. 2:**
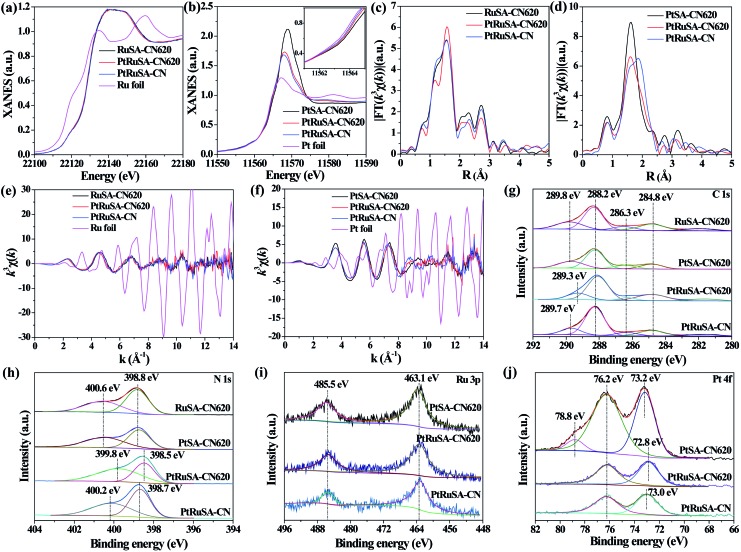
(a) Ru K-edge and (b) Pt L_3_-edge XANES spectra of as-prepared samples and the corresponding *k*^3^-weighted FT spectra of Ru and Pt at (c and d) *R* space and (e and f) *k* space. XPS survey spectra of (g) C 1s, (h) N 1s, (i) Ru 3p and (j) Pt 4f of as-prepared samples.

To explore the relationship between the coordination structure and electronic properties of Pt–Ru monomers, X-ray photoelectron spectroscopy (XPS) was used to analyze the chemical state of Pt–Ru monomers and C_3_N_4_. In the C 1s spectrum, a dominant C 1s peak at 288.2 eV corresponds to the three-coordinated C_3C_ groups (Fig. 2g).^22^ The weak peak at binding energy larger than 288.2 eV is attributed to the C species binding with metal or O atoms. Especially, the peak at 289.3 eV in PtRuSA–CN620 is lower than those in RuSA–CN620 (289.8 eV), PtSA–CN620 (289.8 eV) or PtRuSA–CN (289.7 eV). This suggests that the coexistence of Pt–Ru and rich N vacancies synergistically improves the electron density of partial C atoms. This synergistic effect also appears in the N 1s spectrum. A remarkably lower peak at 399.8 eV appears in PtRuSA–CN620 compared to those in RuSA–CN620 (400.6 eV), PtSA–CN620 (400.6 eV) or PtRuSA–CN (400.2 eV), associated with N_2C_ (Fig. 2h).^22^,^24^ Besides, the peak at 398.5 eV in PtRuSA–CN620, corresponding to N_3C_, also shows a slight red shift compared to those in other samples. In the Ru 3p spectrum, no obvious difference occurs in all samples (Fig. 2i). However, the Pt 4f spectrum exhibits some diversity in PtSA–CN620, PtRuSA–CN620 and PtRuSA–CN (Fig. 2j). Especially, a relatively high peak at 78.8 eV is only observed in PtSA–CN620, corresponding to the oxidized Pt species. However, the addition of Ru removes this peak in PtRuSA–CN620 and PtRuSA–CN, implying the promotion effect of Ru on the reduction of Pt. Besides, the peak at 72.8 eV in PtRuSA–CN620 is lower than that (73.0 eV) in PtRuSA–CN, further confirming the lower chemical valence of Pt in PtRuSA–CN620, which is consistent with the above XANES. Combining the results of C and N 1s spectra, it is speculated that the Pt–Ru monomers on the N_2C_ and C_2C_ sites of PtRuSA–CN620 build an electron-rich region.

To illustrate the origination of the relationship between the coordination structure and electronic properties of neighboring Pt–Ru monomers at the atomic level, density functional theory (DFT)-based theoretical simulation was conducted (Fig. 3). As a comparison, the neighboring Ru–Ru/Pt–Pt and isolated Ru/Pt monomers are also considered. Based on the coordination environment of Pt and Ru, nine models are simulated, denoted as C–Ru–Ru–N, C–Pt–Pt–N, C–Pt–Ru–N, C–Ru–Pt–N, N–Pt–Ru–N, N–Ru–N, C–Ru–N, N–Pt–N and C–Pt–N, respectively (Fig. 3a–i). The calculated results confirm that the Pt–Ru monomers with the coordination structure of C–Pt–Ru–N is more stable than the Ru–Ru/Pt–Pt monomers, isolated Ru/Pt atoms or other Pt–Ru monomers, which is attributed to their more negative adsorption energy (*E*_ads_) by forming two Pt–C, one Pt–N and three Ru–N bonds. Moreover, the formation energy of the C–Pt–Ru–N structure is remarkably 4.9 eV more negative than that of N–Pt–Ru–N (Fig. 3e), suggesting the important role of N vacancies in stabilizing the Pt–Ru monomers. Furthermore, the corresponding projected density of states (PDOS) of the above structures was calculated to investigate their electronic properties. The results show that the 5d states of Pt in C–Pt–Ru–N are located in the most negative region compared to those in the other structures (Fig. 3j). This means that the PtSA in C–Pt–Ru–N is an electron-rich center. However, the 4d states of Ru show relatively less shift with the change in its coordination structure. This finding well explains the results of XAFS and XPS. This electron-rich effect in the C–Pt–Ru–N structure is probably beneficial to some reduction-dependent catalytic reactions, such as the catalytic CO oxidation with O_2_ as the oxidizing agent.

**Fig. 3:**
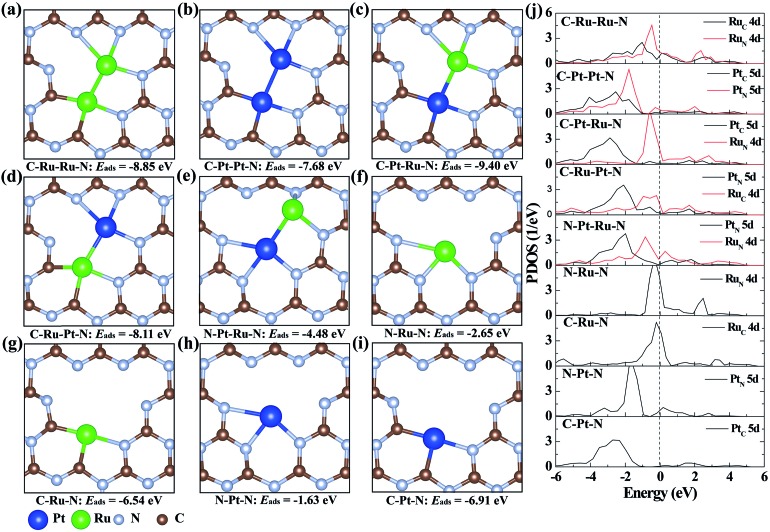
The geometry structures of (a) C–Ru–Ru–N, (b) C–Pt–Pt–N, (c) C–Pt–Ru–N, (d) C–Ru–Pt–N, (e) N–Ru–Ru–N, (f) N–Ru–N, (g) C–Ru–N, (h) N–Pt–N and (i) C–Pt–N in CN. (j) The corresponding projected density of state (PDOS) plots. The dashed line stands for the Fermi level. Both Ru–Ru and Pt–Pt monomers are coordinated with four N and two C atoms. The Pt–Ru monomers are located in three coordination environments: (c) Ru coordinated with three N atoms and Pt coordinated with two C and one N atoms, (d) Pt coordinated with three N atoms and Ru coordinated with two C and one N atoms and (e) Ru coordinated with three N atoms and Pt coordinated with three N atoms.

The catalytic CO oxidation, performed in a continuous flow fixed bed quartz microreactor, was used to reveal the synergistic effect between neighboring Pt–Ru monomers.^30^ The results show that PtRuSA–CN620 possesses a lowest conversion temperature (*T*_100%_) of 150 °C compared to RuSA–CN620, PtSA–CN620, PtRuSA–CN and PtRuNP–CN620 (Fig. 4a). This suggests the synergistic promotion effect of Pt–Ru monomers and N vacancies on the catalytic CO oxidation, which is attributed to the active C–Pt–Ru–N structure. A stability test with PtRuSA–CN620 was carried out by a continuous reaction at 150 °C for 3000 min (Fig. S8†), which shows the good stability of PtRuSA–CN620. The XAFS characterization of PtRuSA–CN620 after the reaction indicates the atomic dispersion of Pt and Ru (Fig. S9†). Besides, the peaks between 2 and 3 Å in Fig. S9a and b† show the existence of Pt–Ru monomers, confirming its high stability. The corresponding HRTEM image confirms that no obvious particles appear in PtRuSA–CN620 after the reaction (Fig. S10†), which also implies the stability of PtRuSA–CN620.

**Fig. 4:**
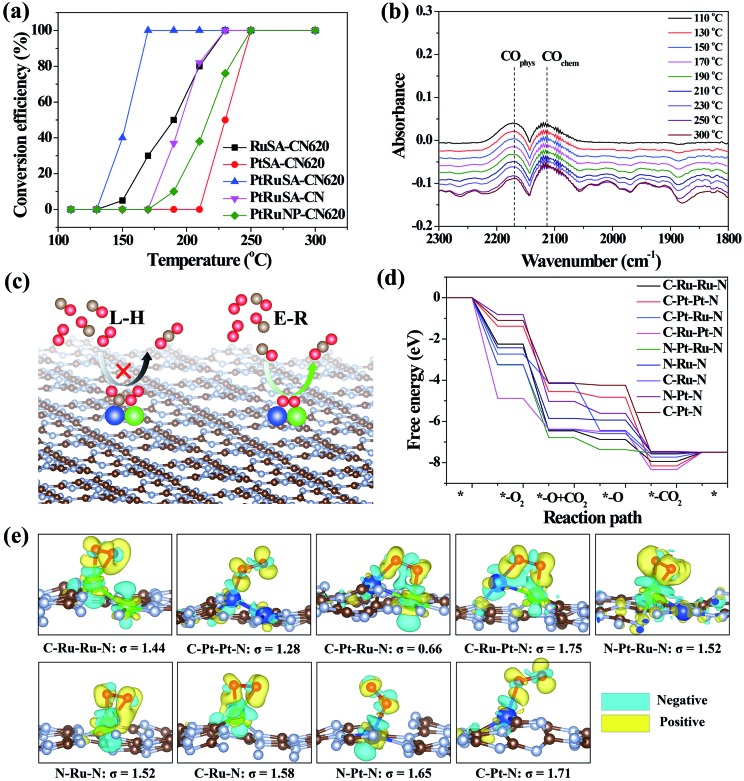
(a) CO conversion as a function of temperature over the as-prepared samples at CO concentration = 1 vol%. (b) The *in situ* infrared spectra of CO oxidation on PtRuSA–CN620. (c) Reaction mechanisms of CO oxidation. (d) The free energy pathways of catalytic CO oxidation on the C–Ru–Ru–N, C–Pt–Pt–N, C–Pt–Ru–N, C–Ru–Pt–N, N–Pt–Ru–N, N–Ru–N, C–Ru–N, N–Pt–N and C–Pt–N structures. (e) The geometry structures and charge density difference mappings of O_2_ adsorptions. The overall geometry structures of CO oxidation pathways are shown in Fig. S13.† The sky blue and yellow isosurfaces stand for the negative and positive charges, respectively. The isosurface of charge density is set to 0.005 e Å^–3^ in all figures.

*In situ* DRIFTS measurement was further performed to determine the catalytic reaction mechanism on different samples (Fig. 4b). The results reveal that the CO adsorption only shows two weak stretching vibration peaks at 2171 and 2111 cm^–1^ on PtRuSA–CN620 in the catalytic reaction, corresponding to the physical and chemical adsorption of CO, respectively.^5^,^29^ In comparison, two similar peaks also appear in pure CN620 and PtSA–CN620 (Fig. S11†). Though the ratio of chemical CO adsorption of PtSA–CN620 to physical CO adsorption is slightly higher than that of pure CN620, the improved chemical CO adsorption on PtSA–CN620 is far weaker than those in the previous literature.^5^,^31^,^32^ Hence, the shown CO adsorption on PtSA produces little effect on the catalytic reaction since most PtSAs are free of CO adsorption. This suggests that the CO adsorption is independent of Pt or Ru in the catalytic reaction. Furthermore, the stability of CO adsorption on PtRuSA–CN620 was examined, revealing that CO adsorption on PtRuSA–CN620 is weak (Fig. S12†). Hence, CO adsorption in its catalytic oxidation is not dominant. In general, the catalytic CO oxidation follows two mechanisms: Langmuir–Hinshelwood (L–H) and Eley–Rideal (E–R) mechanisms (Fig. 4c).^31^ In the L–H mechanism, both CO and O_2_ are adsorbed on the catalyst surface, such as Pt-loaded TiO_2_. The corresponding IR observation should obtain an obvious CO adsorption peak. However, the above IR observation does not support this mechanism. For the E–R mechanism, only CO or O_2_ is adsorbed on the catalyst surface. Hence, the catalytic CO oxidation on the as-prepared samples can be attributed to the E–R mechanism where the CO adsorption is not necessary. Besides, it is speculated that the O_2_ activation plays an important role in the catalytic CO oxidation.

To explain the high activity of PtRuSA–CN620, DFT simulation on the catalytic CO oxidation process was performed. Considering the structural features of the as-prepared samples, the simulations were based on the above C–Ru–Ru–N, C–Pt–Pt–N, C–Pt–Ru–N, C–Ru–Pt–N, N–Pt–Ru–N, N–Ru–N, C–Ru–N, N–Pt–N and C–Pt–N structures (Fig. S13†). The results show that all reaction steps except the final CO_2_ desorption step are exothermic (Fig. 4d). For a catalytic reaction, an excessive energy decrease in one step is often not beneficial to the whole reaction with respect to thermodynamics, which easily causes a high reaction barrier or less negative reaction energy in the other steps. Thus balancing the energy decrease in the reaction steps is significant for the catalytic reaction. Here we use a standard deviation (*σ*) method to evaluate the catalytic CO oxidation activity of the above structures (details in the Theoretical simulation of the ESI†). In this method, the smaller standard deviation among the reaction energies of different steps endows the corresponding structure with higher catalytic activity. The fore four exothermic steps, including O_2_ adsorption, CO oxidation by the adsorbed O_2_ and O species and the first CO_2_ desorption are considered. The standard deviations of reaction step energies in C–Ru–Ru–N, C–Pt–Pt–N, C–Pt–Ru–N, C–Ru–Pt–N, N–Pt–Ru–N, N–Ru–N, C–Ru–N, N–Pt–N and C–Pt–N are calculated to be 1.44, 1.28, 0.66, 1.75, 1.52, 1.52, 1.58, 1.65, and 1.71 eV, respectively (Fig. 4e). These results suggest that the C–Pt–Ru–N structure possesses optimized catalytic reaction steps for CO oxidation. This is originated from the effective activation of O_2_ between Pt–Ru monomers by causing bridge-type O_2_ adsorption with one Pt–O bond and one Ru–O bond (Fig. 4e). In this O_2_ adsorption, the electrons (sky blue region) can be effectively aggregated between two O atoms, which contributes to the activation of O_2_. However, on C–Ru–Ru–N and C–Pt–Pt–N structures, the O_2_ activation only occurs on one RuSA or PtSA, corresponding to a less negative reaction energy. Though one Pt–O bond and one Ru–O bond can simultaneously form in the O_2_ activation on the C–Ru–Pt–N structure, the reaction energy is excessively negative, leading to the low oxidizing activity of the formed O species. On the N–Pt–Ru–N structure, the reaction energy of the first CO oxidation step is also excessively negative. Besides, the left O species on the RuSA is less active, which leads to a less negative reaction energy in the second CO oxidation step. As for the isolated Ru or Pt atoms, the bridge-type O_2_ adsorption is not observed. Hence, the DFT results confirm that the best site for the catalytic CO oxidation is the C–Pt–Ru–N structure. According to the above experimental analysis, PtRuSA–CN620 possesses more C–Pt–Ru–N structures. Thus the theoretical simulations well explain the highest catalytic CO oxidation activity of PtRuSA–CN620 compared to the other samples.

## Conclusions

To summarize, we constructed neighboring Pt–Ru monomers on a N-vacancy-rich g-C_3_N_4_ surface by an icing-assisted photocatalytic reduction method. Aberration-corrected-STEM and XAFS confirms the existence of neighboring Pt–Ru monomers in the N_2C_ and C_2C_ sites of N-vacancy-rich g-C_3_N_4_. Using DFT simulations, an electron-rich C–Pt–Ru–N structure was defined as not only a stable thermodynamic model of neighboring Pt–Ru monomers, but also an optimized site for the catalytic CO oxidation compared to neighboring Ru–Ru/Pt–Pt monomers or isolated Ru/Pt atoms. This finding demonstrates an opportunity for developing a new class of atomically dispersed metal catalysts.

## Conflicts of interest

There are no conflicts to declare.

## Supplementary Material

Supplementary information
