# Supplementary material for: Synergetic interaction between neighboring platinum and ruthenium monomers boosts CO oxidation
Source: Chem Sci. 2019 Apr 25;10(23):5898–905. doi: 10.1039/c9sc00658c (PMC6566076; doi:10.1039/c9sc00658c)
Supplement: Supplementary file 1 [file SC-010-C9SC00658C-s001.pdf]

Electronic Supplementary Material (ESI) for Chemical Science.
This journal is © The Royal Society of Chemistry 2019

Supporting Information

Synergetic interaction between neighboring platinum and ruthenium monomers boosts CO oxidation

Peng Zhou,[a] Xingang Hou,[b] Yuguang Chao,[a] Wenxiu Yang,[a] Weiyu Zhang,[a] Zijie Mu,[a] Jianping Lai,[a] Fan Lv,[a] Yang Kuan,[c] Yuxi Liu,[c] Jiong Li,[d] Jingyuan Ma,[d] Jun Luo[b] and Shaojun Guo*[a,e,f]

[a]Department of Materials Science and Engineering, Peking University, Beijing 100871, China.

[b]Center for Electron Microscopy, Tianjin Key Laboratory of Advanced Functional Porous Materials, Institute for New Energy Materials & Low-Carbon Technologies, School of Materials, Tianjin University of Technology, Tianjin 300384, China.

[c]Laboratory of Catalysis Chemistry and Nanoscience, Department of Chemistry and Chemical Engineering, College of Environmental and Energy, Engineering, Beijing University of Technology, Beijing 100124, China.

[d]Shanghai Synchrotron Radiation Facility, Shanghai Institute of Applied Physics, Chinese Academy of Sciences, Shanghai 201204, China.

[e]The Beijing Innovation Center for Engineering Science and Advanced Technology, Peking University, Beijing 100871, China.

[f]Key Laboratory of Theory and Technology of Advanced Batteries Materials, College of Engineering, Peking University, Beijing 100871, China, E-mail: guosj@pku.edu.cn.

EXPERIMENTAL SECTION

Chemicals. All of the reagents were of analytical grade, and used without further purification. H_2PtCl_6, $RuCl_3$ and dicyandiamide were purchased from Aladdin (Shanghai, China).

Synthesis of *g*-C_3N_4. Deionized (DI) water was used in all experiments.*g*-C_3N_4 photocatalysts were prepared by following a typical thermal polymerization procedure. Briefly, 5 g of dicyandiamide was put into a covered crucible, heated to 500 °C at a ramp rate of 5 °C min^{-1} in a tube furnace under air condition and then maintained at this temperature for additional 4 h. After being cooled down to room temperature, the resultant powders were grinded and collected, noted as CN.

Synthesis of N-vacancy-riched *g*-C_3N_4. 1 g of *g*-C_3N_4 was put into a crucible, heated to 620 °C at a ramp rate of 5 °C min^{-1} in a tube furnace under N_2 condition, respectively, and maintained at the corresponding temperature for 2 h. After being cooled down to room temperature, the resultant powders were collected, noted as CN620.

Synthesis of PtRuNP-loaded CN620 (PtRuNP-CN620). For another comparison, PtRuNP-CN620 was synthesized by directly irradiating the mixed solution of CN620 and H_2PtCl_6/$RuCl_3$ without the pre-treatment of liquid nitrogen. Other procedures were similar to the above preparation method for making the PtRuSA-CN620.

Synthesis of PtRuSA-CN620. 0.04 g of CN620 was dispersed in 10 mL of deionized water containing 2 μmol H_2PtCl_6 and 1 μmol $RuCl_3$ under magnetic stirring for 30 min. Next, the mixed solution was ultrasonicated for 30 min, and stirred for 12 h. Then, the mixed solution was rapidly frozen by liquid nitrogen, followed by irradiating under a 300 W Xe light with the light filter of 420 nm. After 10 min irradiation, the ice layer was naturally melted. The formed solution was centrifugalized and separated. The obtained precipitates were further washed with deionized water for two times. Finally, the precipitates were dried in an oven at 60 °C for 12 h. Similarly, the RuSA-loaded CN620 (RuSA-CN620) and PtSA-loaded CN620 (PtSA-CN620) were prepared by using 4 μmol H_2PtCl_6 and 2 μmol $RuCl_3$ as the precursors, respectively. As a comparison, the PtRuSA-loaded CN (PtRuSA-CN) was prepared by using CN as the support of PtRuSA.

Catalyst characterization. The X-ray diffraction (XRD) patterns, obtained on an X-ray diffractometer (Rigaku, Japan) using Cu Kα radiation at a scan rate of 0.05° 2θ s^{-1}, were used to characterize the crystalline phase of the samples. The accelerating voltage and applied current were 40 kV and 80 mA, respectively. Aberration-corrected HAADF-STEM analysis was

conducted on JEM-ARM200F transmission electron microscope and FEI Tecnai G2 F20 S-Twin HRTEM working at 300 kV. X-ray photoelectron spectroscopy (XPS) measurements were performed on an ESCALAB 250Xi electron spectrometer with Mg Kα (1253.6 eV) source. All binding energies were referenced to the C 1s peaks at 284.8 eV from the adventitious carbon. The content of Pt elements in the as-prepared samples was analysed by an inductively coupled plasma-atomic emission spectrometer (ICP-AES) on PerkinElmer Optima 7300DV.

XAFS measurement and data analysis. XAFS spectra at the Pt L_3-edge and Ru K-edge were measured at the BL14W1 beamline of the Shanghai Synchrotron Radiation Facility (SSRF) at 3.5 GeV with a maximum current of 300 mA, China. The hard X-ray was monochromatized with Si(311) double-crystal monochromator. The acquired EXAFS data were processed according to the standard procedures using the ATHENA module of the IFEFFIT software packages. The k^3-weighted $\chi(k)$ data in the k-space ranging from 2.0-12.4 $Å^{-1}$ were Fourier transformed to real (R) space using a hanning windows (dk = 1.0 $Å^{-1}$) to separate the EXAFS contributions from different coordination shells. To obtain the detailed structural parameters around Pt and Ru atom in the as-prepared samples, the quantitative curve-fittings were carried out for the Fourier transformed $k^3\chi(k)$ in the R-space using the ARTEMIS module of IFEFFIT3. Effective backscattering amplitudes F(k) and phase shifts Φ(k) of all fitting paths were calculated by the *ab initio* code FEFF8.0. During the fitting analysis, the amplitude reduction factor S_0^2 was fixed to the best-fit value of 0.90, which was determined from fitting the reference sample of metal Pt bulk, PtN_2 bulk, PtC bulk, Ru bulk, RuN_2 bulk and RuC bulk. In general, it was difficult to distinguish Pt/Ru-O and Pt/Ru-N coordination. Thus the Pt/Ru-N coordination was used to describe the Pt/Ru-N/O coordination. To fit the data of PtSA-CN620, RuSA-CN620, PtRuSA-CN620 and PtRuSA-CN, the interatomic distance (R) and the Debye-Waller factor (σ^2) were allowed to vary. We have distinguished Pt/Ru-N/O and Pt/Ru-C from Pt-Pt/Ru-Ru coordination, considering the existing bonding length difference between them.

Catalytic activity measurements. A continuous flow fixed bed quartz microreactor (diameter = 4 mm) was employed to evaluate the catalytic activities of the samples at atmospheric pressure for the oxidation of CO and toluene. The reactant feedstocks were: (1) 1 vol% CO + 20 vol% O_2 + N_2 (balance), total flow rate = 10 mL min^{-1}, and space velocity (SV) = ca. 12000 mL g^{-1} h^{-1}. The inlet and outlet CO concentrations were analysed online by a gas chromatograph (Shimadzu GC-14C) equipped with a TCD detector and a Carboxen 1000 column for CO analysis. To real-time monitoring the activity in the stability test, an online mass spectrometer (QMG220M2, detector: C-SEM/Faraday, Pfeiffer) was used to analyse the reactant CO and product CO_2.

***In-situ* DRIFTS.** The *In-situ* DRIFTS of CO oxidation on the as-prepared samples was performed on a Thermo Nicolet 6700 FT-IR spectrometer equipped with a mercury cadmium telluride (MCT) detector. The reaction system consisted of a praying mantis diffuse reflectance accessory and a reaction cell equipped with a heater (Harrick Scientific). 50 mg of samples were housed at a sample cup inside the reaction cell. A cover dome contained three windows: two were made of ZnSe to permit the entry and exit of detection infrared beam and the third (quartz) was for the transmission of UV-light beam during *in situ* reactions. Before IR measurement, the samples were heated at 300 ^{o}C for 30 min under air flow (100 mL min^{-1}) to clear surface. After that, all of the pretreated samples were flushed by a 1 vol% CO + 20 vol% O_2 + N_2 (balance), total flow rate = 10 mL min^{-1} at different reaction temperatures. The corresponding IR spectra were collected. The spectra were converted to Kubelka-Munk unit using Omnic™ software when used for quantification.

Theoretical simulation. The geometry structures and electronic properties of Ru-Ru, Pt-Pt and Pt-Ru monomers on *g*-C_3N_4 were investigated by the density functional theory (DFT) calculations based on the VASP package using the PBE exchange-correlation function. The interaction between valence electrons and the ionic core was described by the PAW pseudo-potential. The model of *g*-C_3N_4 was simulated by a periodic atomic layer containing 24 C atoms and 32 N atoms. The N vacancy was built by removing a N_{2C} atom. The geometry structures of pure and N-vacancy-modified *g*-C_3N_4 were optimized with the cutoff of 400 eV. All the atoms in the model were allowed to adjust until the magnitude of all residual forces was less than 0.001 eV Å^{-1}. Considering the calculation cost, the geometry optimization was only performed at Gamma point. The geometry structures of different reaction steps were also obtained by the same optimization setting. After the geometry optimization, the PDOS was calculated by the cutoff energy of 400 eV and the Monkhorst-Pack k-point mesh of 2 × 2 × 1. The adsorptions of Ru-Ru, Pt-Pt and Pt-Ru monomers at the low-coordinated C_{2C} and N_{2C} sites were considered. The adsorption energy (E_{ads}) of Ru-Ru, Pt-Pt and Pt-Ru monomers on *g*-C_3N_4 was used to evaluate the stability of coordination structure, which was calculated by the following formula:

$$E_{ads} = E_{CN+Pt/Ru-Pt/Ru} - E_{CN} - E_{Pt/Ru-Pt/Ru} \quad (1)$$

where $E_{CN+Pt/Ru-Pt/Ru}$ was the total free energy of Pt/Ru-Pt/Ru-adsorbed CN, E_{CN} was the free energy of CN and $E_{Pt/Ru-Pt/Ru}$ was the free energy of Pt/Ru-Pt/Ru monomers in vacuum.

The simulation on the catalytic CO oxidation was performed by the E-R mechanism according to the experimental observations. The geometry structures of all reaction steps were optimized by

the cutoff of 400 eV until the magnitude of all residual forces was less than 0.001 eV Å^{-1}. A standard deviation method was used to evaluate the catalytic CO oxidation activity of above structures. In this method, the standard deviation (σ) was calculated by the following formula:

$$\sigma = \sqrt{\frac{1}{N}\sum_{i=1}^{N}(x_i - \mu)^2} \qquad (2)$$

where N was the number of step, x_i was the reaction energy of step *i* and μ was the average reaction energy. The smaller σ value stands for the less difference among the reaction energies of energy step, which is corresponding to an optimized reaction path.

Figures

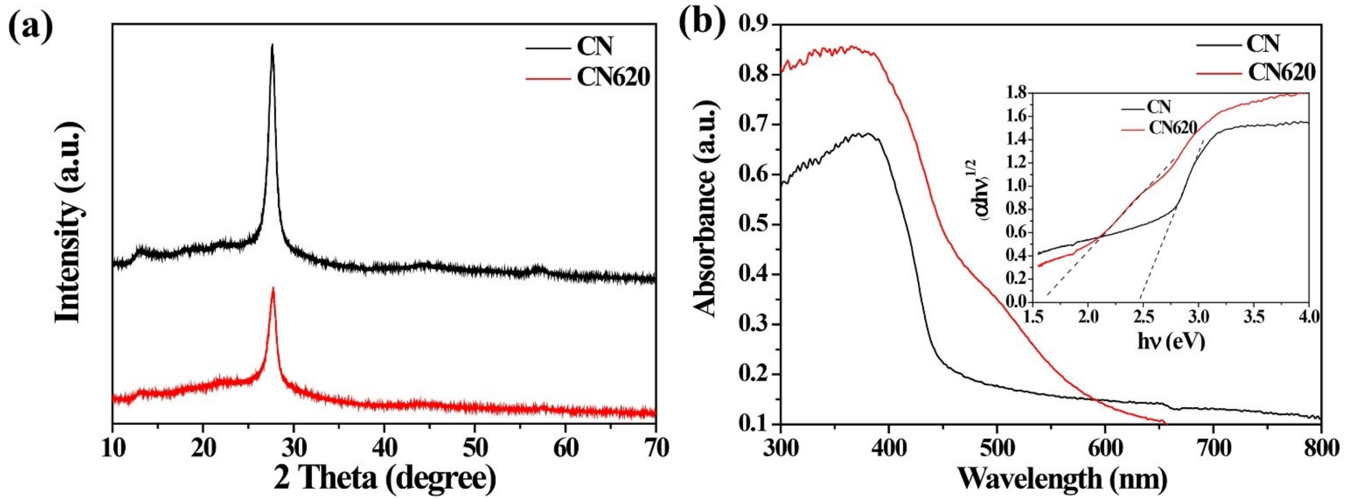

Figure S1. (a) XRD and (b) DRS of CN and CN620.

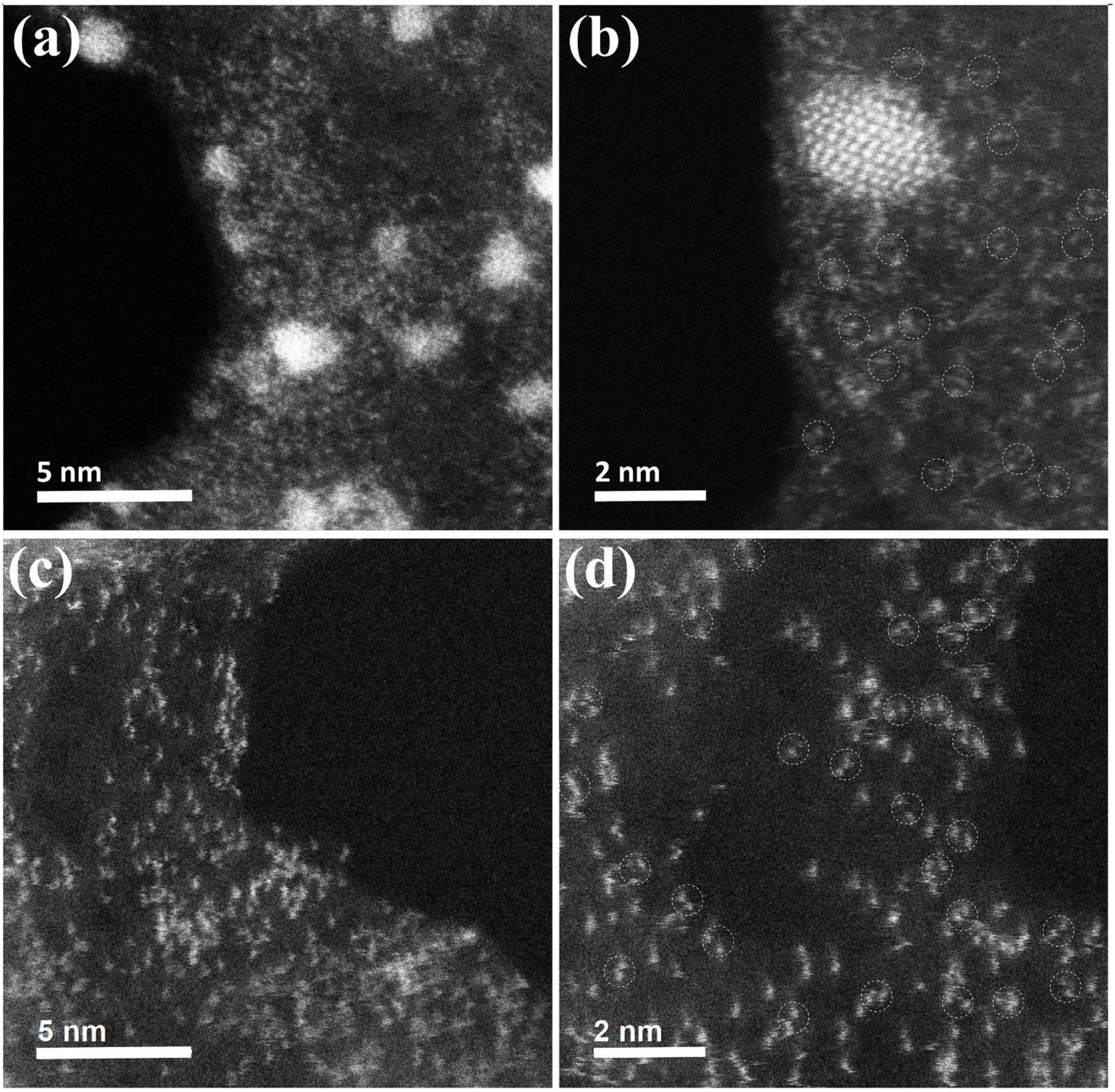

Figure S2. HAADF-STEM images of (a, b) RuSA-CN620 and (c, d) PtSA-CN620. RuSA-CN620 and PtSA-CN620 were characterized on FEI Tecnai G2 F20 S-Twin and JEM-ARM200F transmission electron microscope working at 300 kV, respectively.

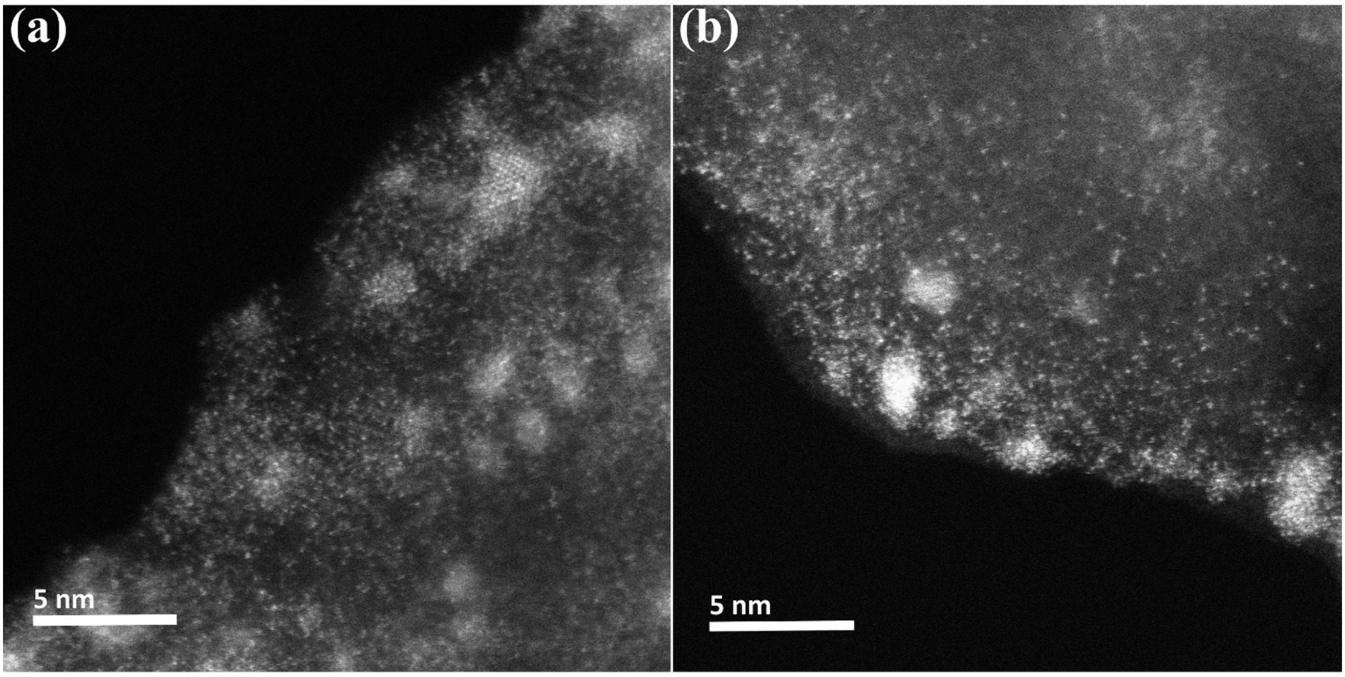

Figure S3. HAADF-STEM image of PtRuSA-CN.

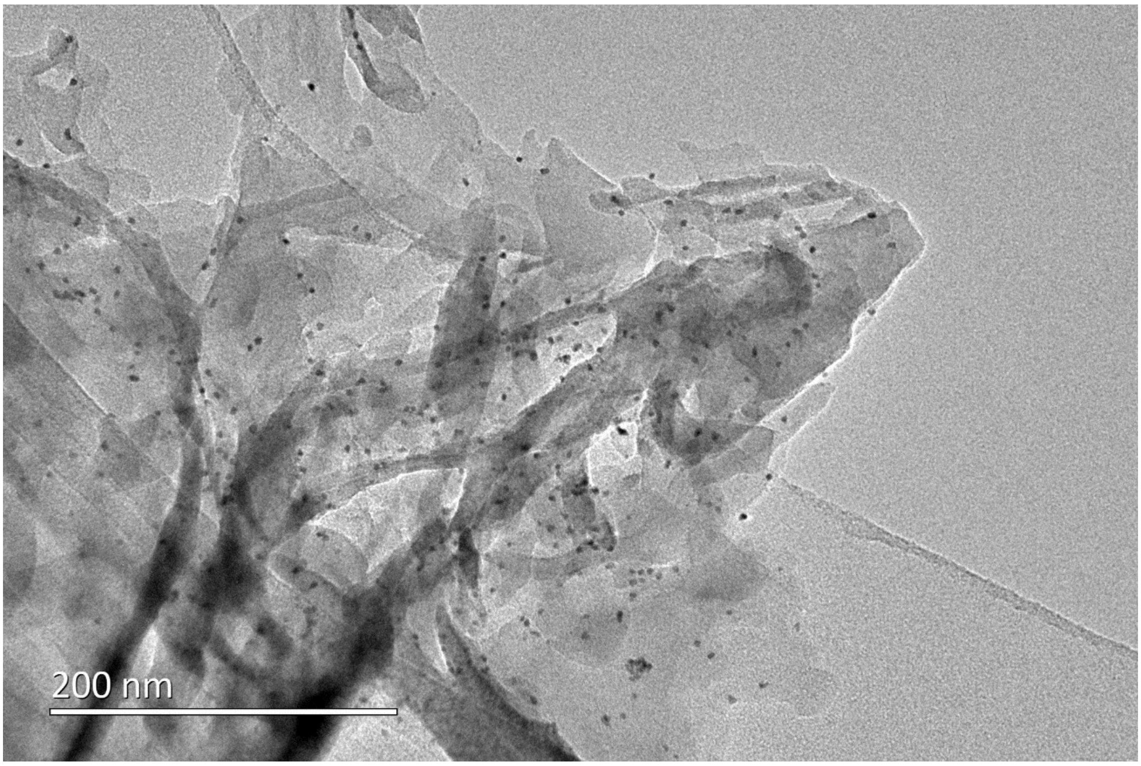

Figure S4. HRTEM image of PtRuNP-CN620.

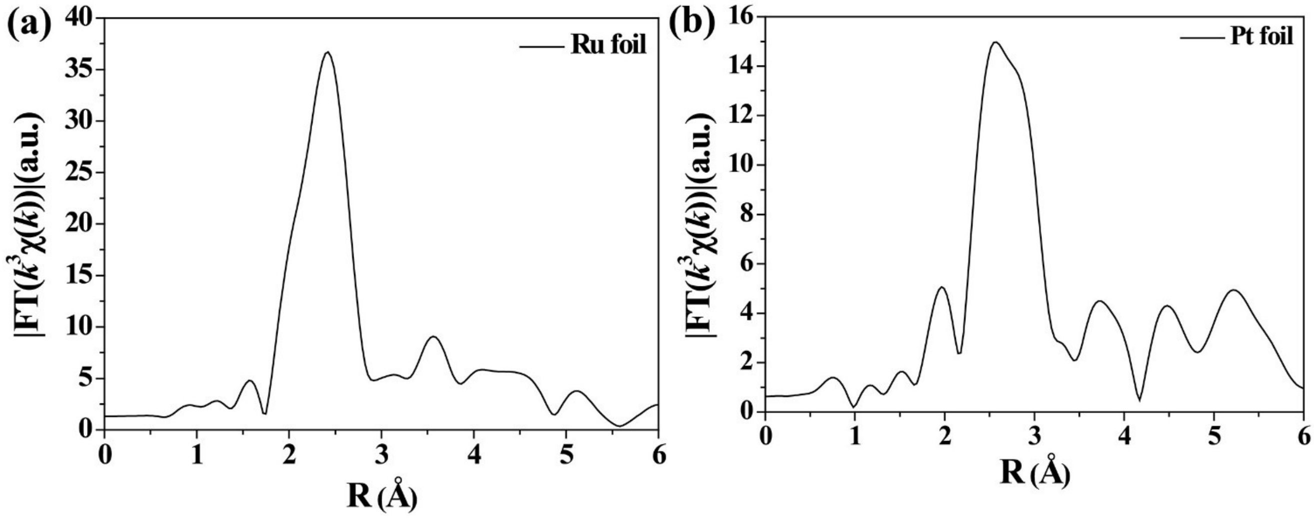

Figure S5. (b) K-edge XANES spectrum of Ru foil and L_3-edge XANES spectrum of Pt foil.

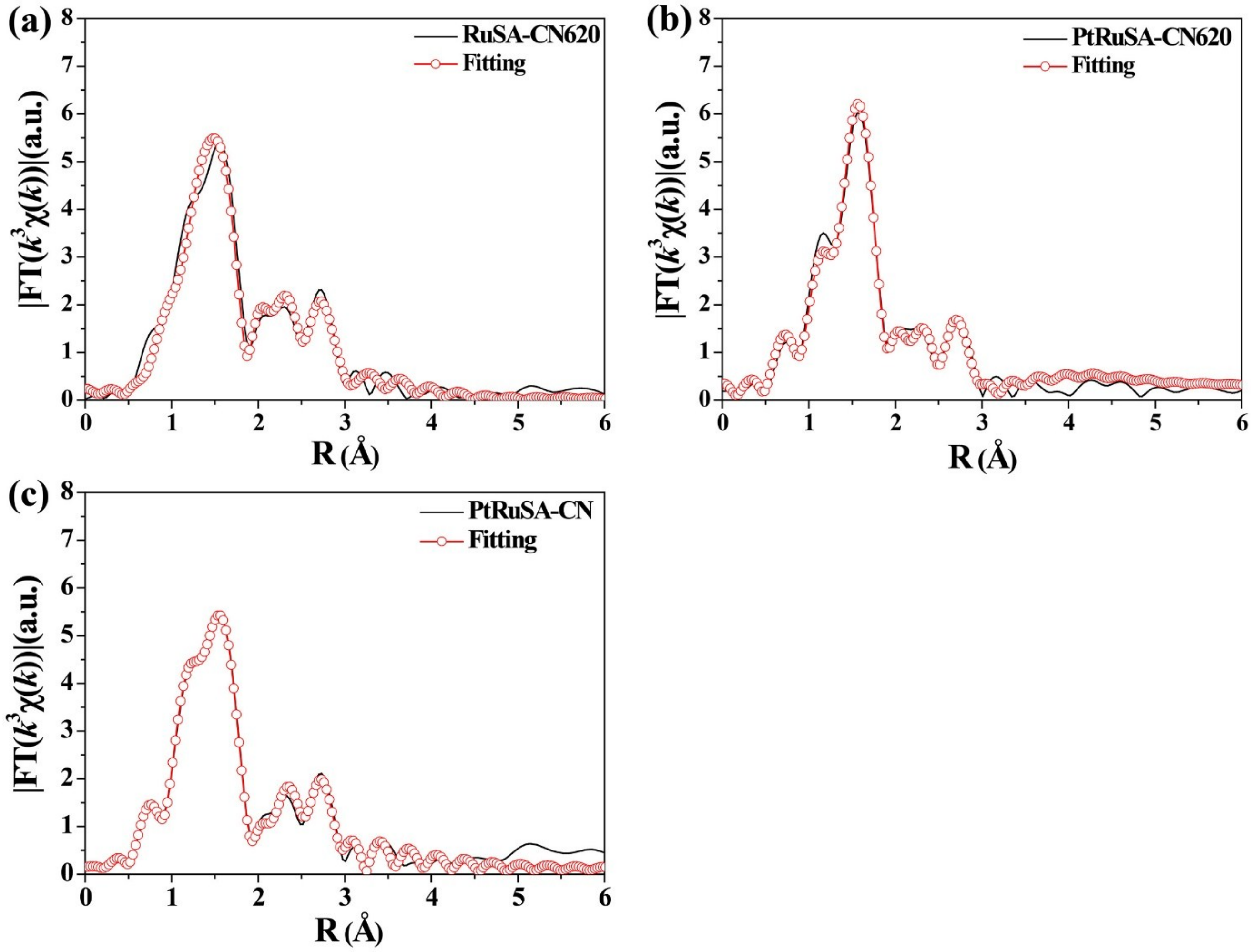

Figure S6. The Ru EXAFS fitting curves of RuSA-CN620, PtRuSA-CN620 and PtRuSA-CN at R space.

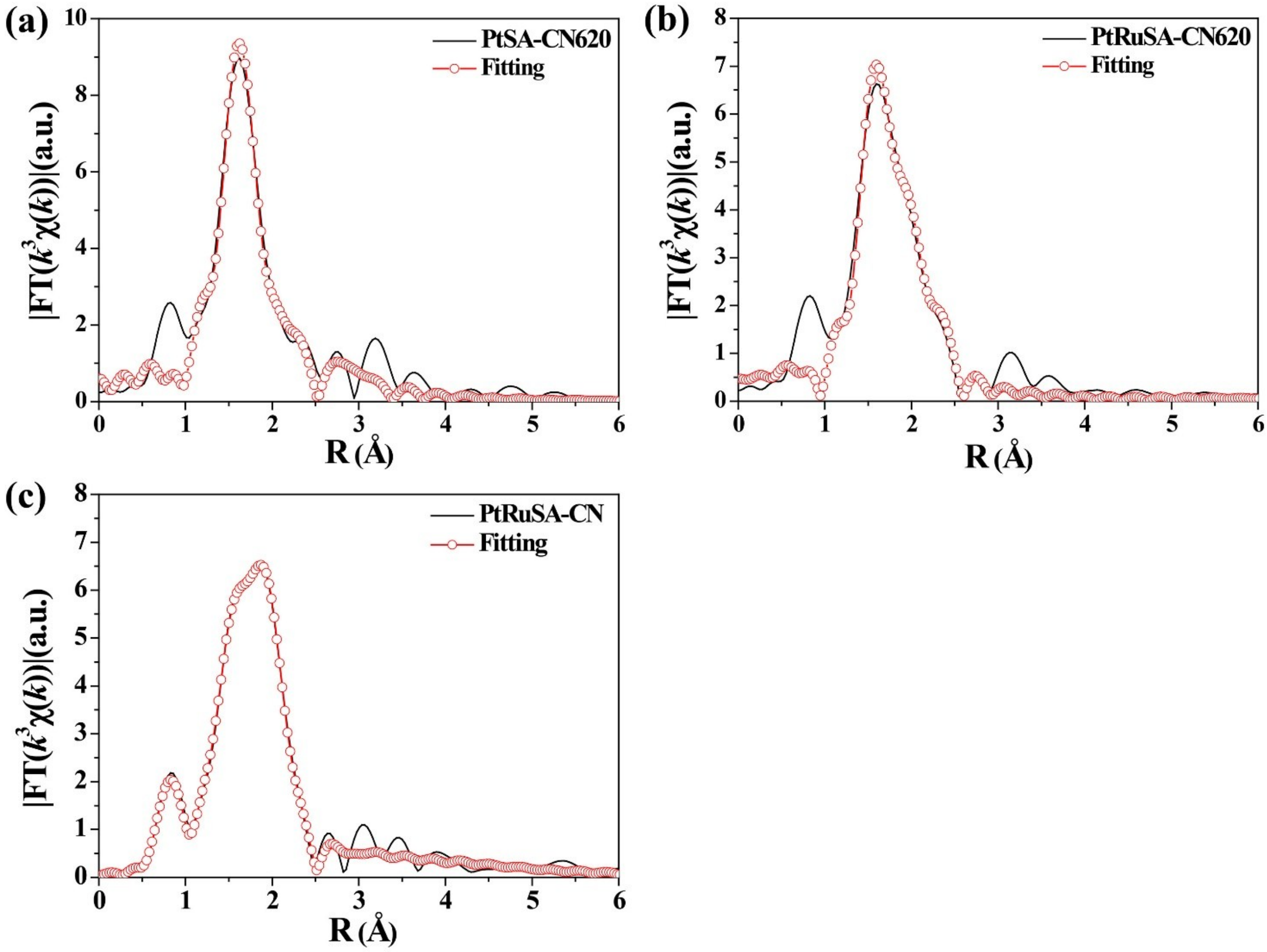

Figure S7. The Pt EXAFS fitting curves of PtSA-CN620, PtRuSA-CN620 and PtRuSA-CN at R space.

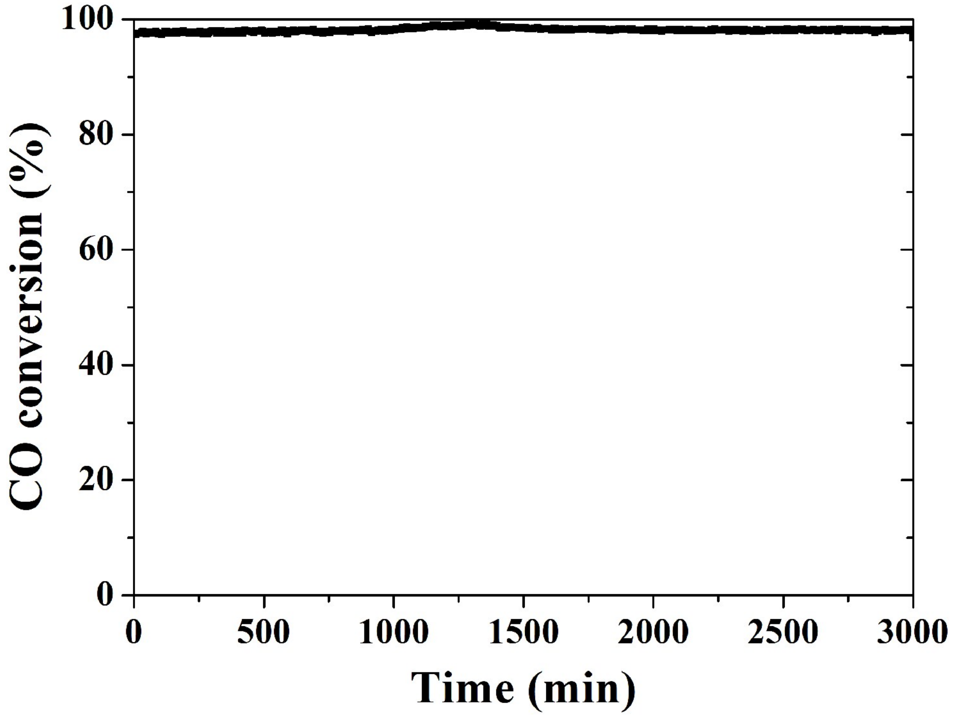

Figure S8. CO conversion as a function of time over the PtRuSA-CN620 sample at 150 ^{o}C.

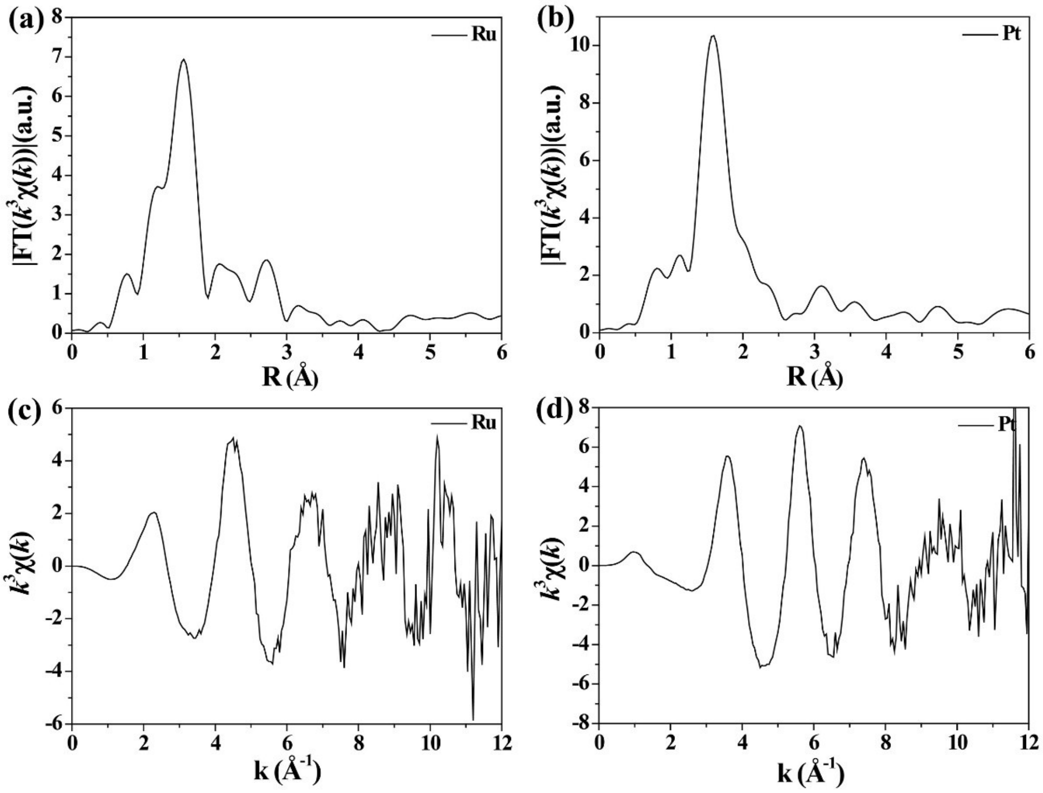

Figure S9. The k^3-weighted FT spectra of Ru and Pt at (a, b) R and (c, d) k space in PtRuSA-CN620 after CO oxidation reaction at 150 ^oC.

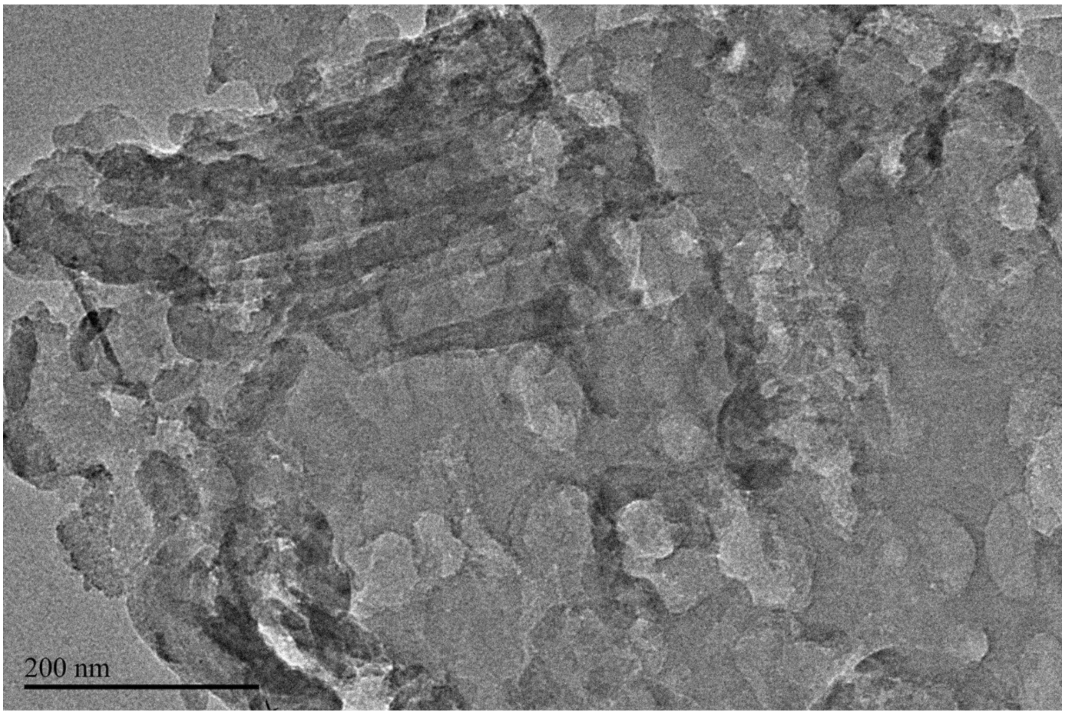

Figure S10. HRTEM image of PtRuSA-CN620 after CO oxidation reaction at 150 ^{o}C.

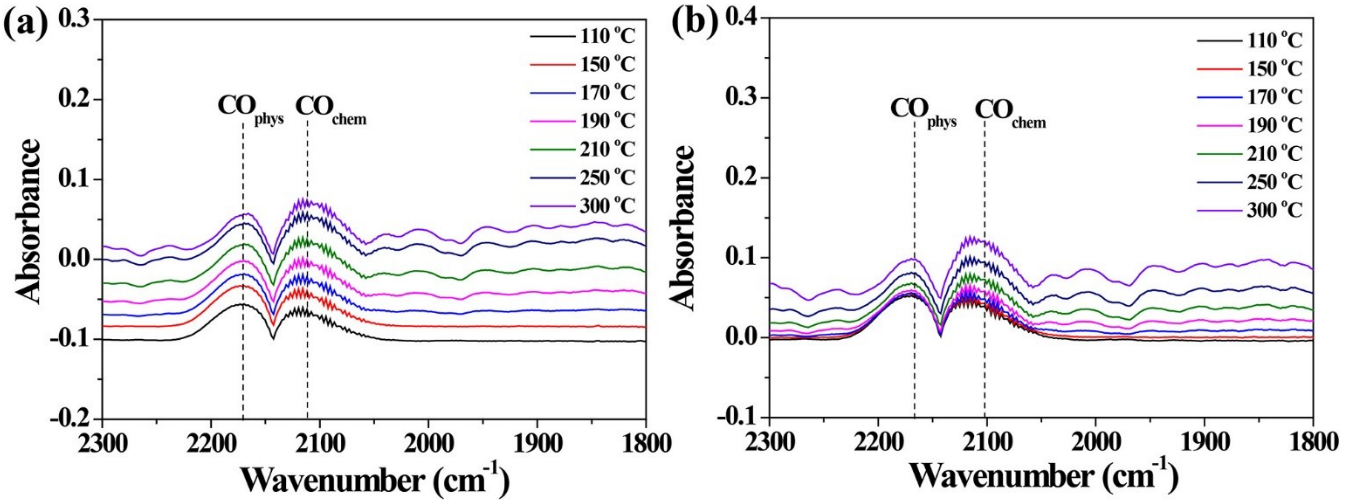

Figure S11. The *in-situ* infrared spectra of CO oxidation on (a) CN620 and (b) PtSA-CN620.

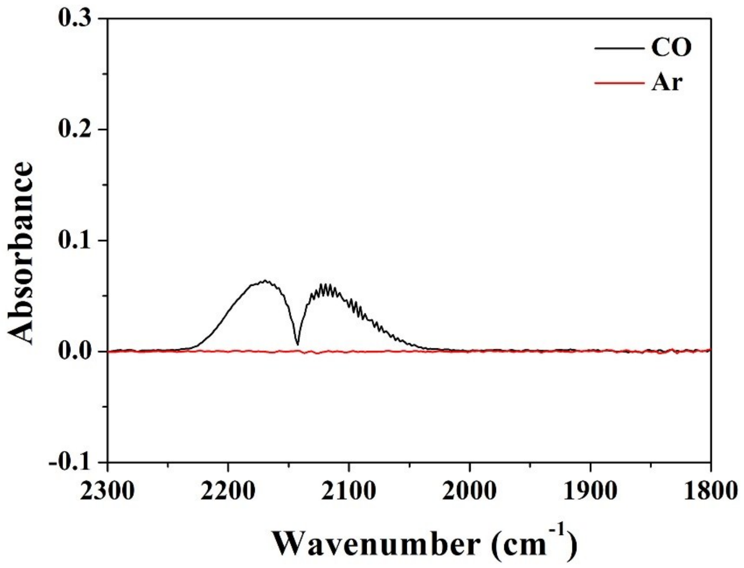

Figure S12. The *in-situ* infrared spectra of CO adsorption on PtRuSA-CN620. The experiment of CO adsorption was performed at 150 ^{o}C. Firstly, CO was adsorbed on PtRuSA-CN620 under a gas flow of 1 vol% CO + 99 vol% Ar. After 10 min adsorption, the CO flow was replaced by the pure Ar flow. The result shows that CO adsorption only occurs in the presence of CO flow, indicating the low stability of CO adsorption on PtRuSA-CN620.

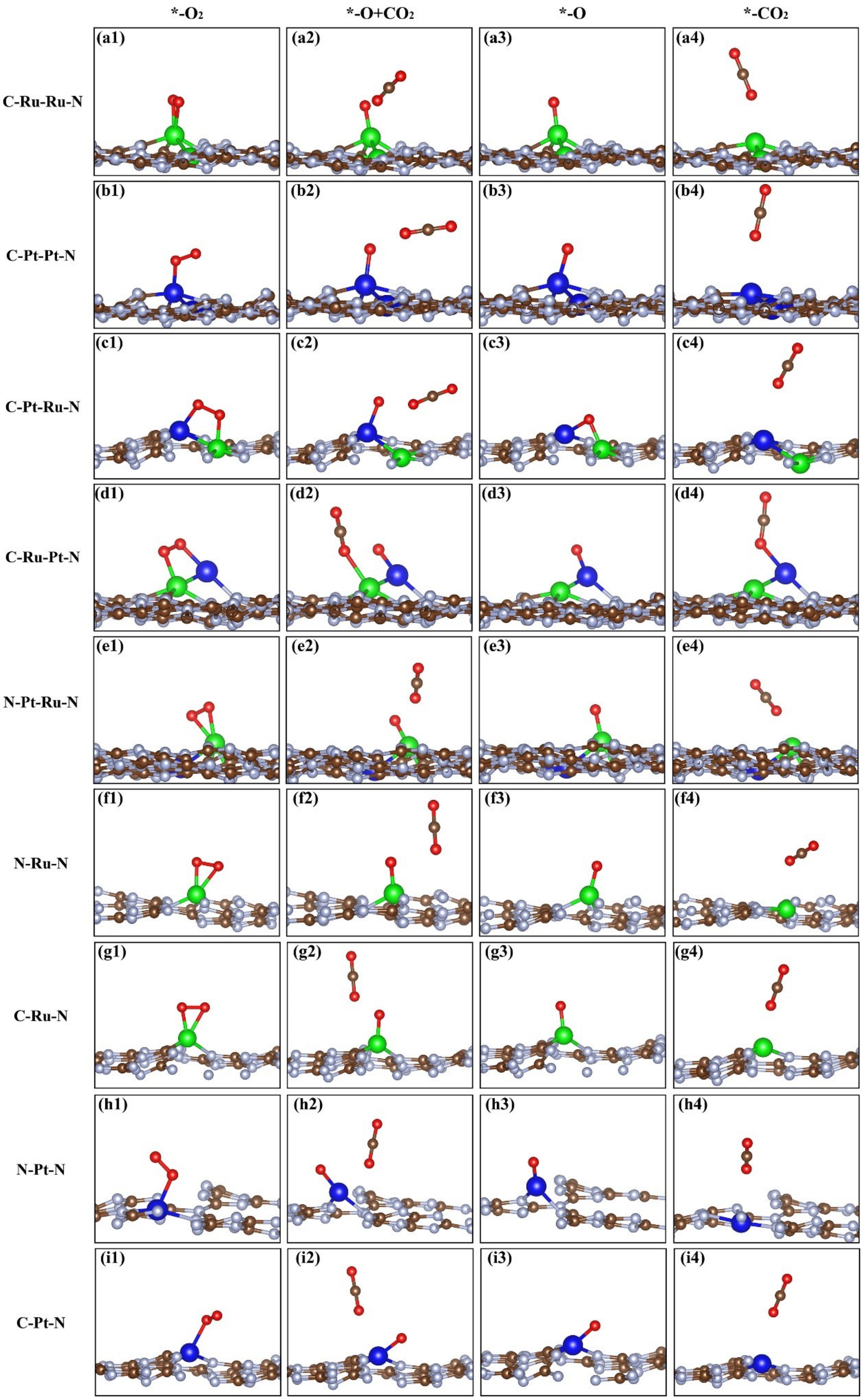

Figure S13. Reaction pathway of CO oxidation on (a) C-Ru-Ru-N, (b) C-Pt-Pt-N, (c) C-Pt-Ru-N, (d) C-Ru-Pt-N, (e) N-Pt-Ru-N, (f) N-Ru-N, (g) C-Ru-N, (h) N-Pt-N and (i) C-Pt-N.

Table S1. The Pt and Ru contents of RuSA-CN620, PtSA-CN620, PtRuSA-CN620, PtRuSA-CN and PtRuNP-CN620 were measured by ICP-OES.

Sample	RuSA-CN620	PtSA-CN620	PtRuSA-CN620	PtRuSA-CN	PtRuNP-CN620
Ru content (wt%)	0.40		0.45	0.32	0.33
Pt content (wt%)		1.34	0.51	0.59	0.54

Table S2. EXAFS fitting results for the Ru in RuSA-CN620, PtRuSA-CN620 and PtRuSA-CN.

Sample	Path	Coordination number	R (Å)	σ^2 (Å^2)	R-factor
RuSA-CN620	Ru-C	0.43	2.45	0.005	0.017
	Ru-N	7.81	2.05	0.010	
	Ru-Ru	1.38	2.88	0.010	
PtRuSA-CN620	Ru-C	3.48	2.33	0.025	0.008
	Ru-N	2.20	2.13	0.005	
	Ru-Ru	0.36	2.85	0.006	
	Ru-Pt	0.72	2.87	0.006	
PtRuSA-CN	Ru-C	2.58	2.09	0.002	0.006
	Ru-N	6.50	1.91	0.016	
	Ru-Ru	0.86	2.72	0.002	
	Ru-Pt	1.42	2.67	0.001	

Table S3. EXAFS fitting results for the Pt in EXAFS fitting results for PtSA-CN620, PtRuSA-CN620 and PtRuSA-CN.

Sample	Path	Coordination number	R (Å)	σ^2 (Å^2)	R-factor
PtSA-CN620	Pt-C	2.02	2.52	0.001	0.008
	Pt-N	5.62	2.04	0.007	
	Pt-Pt	0.84	2.82	0.008	
PtRuSA-CN620	Pt-C	1.29	2.20	0.007	0.017
	Pt-N	3.34	2.02	0.004	
	Pt-Ru	0.98	2.52	0.004	
	Pt-Pt	0.48	2.51	0.004	
PtRuSA-CN	Pt-C				0.005
	Pt-N	4.24	2.08	0.005	
	Pt-Ru	1.34	2.55	0.004	
	Pt-Pt	0.67	2.53	0.004	
